# Interplay of the transcription factor MRTF-A and matrix stiffness controls mammary acinar structure and protrusion formation

**DOI:** 10.1186/s12964-022-00977-2

**Published:** 2022-10-13

**Authors:** Marie-Luise Melcher, Ines Block, Karolin Kropf, Anurag Kumar Singh, Guido Posern

**Affiliations:** Institute for Physiological Chemistry, Medical Faculty, Martin Luther University Halle-Wittenberg, 06114 Halle (Saale), Germany

**Keywords:** Mrtf, Mammary acini, Mechanotransduction, Stiffness, Protrusion formation

## Abstract

**Background:**

Ongoing differentiation processes characterize the mammary gland during sexual development and reproduction. In contrast, defective remodelling is assumed to be causal for breast tumorigenesis. We have shown recently that the myocardin-related transcription factor A (MRTF-A) is essential for forming regular hollow acinar structures. Moreover, MRTF-A activity is known to depend on the biochemical and physical properties of the surrounding extracellular matrix. In this study we analysed the mutual interaction of different matrix stiffnesses and MRTF-A activities on formation and maintenance of mammary acini.

**Methods:**

Human MCF10A acini and primary mature organoids isolated from murine mammary glands were cultivated in 3D on soft and stiff matrices (200–4000 Pa) in conjunction with the Rho/MRTF/SRF pathway inhibitor CCG-203971 and genetic activation of MRTF-A.

**Results:**

Three-dimensional growth on stiff collagen matrices (> 3000 Pa) was accompanied by increased MRTF-A activity and formation of invasive protrusions in acini cultures of human mammary MCF10A cells. Differential coating and synthetic hydrogels indicated that protrusion formation was attributable to stiffness but not the biochemical constitution of the matrix. Stiffness-induced protrusion formation was also observed in preformed acini isolated from murine mammary glands. Acinar outgrowth in both the MCF10A acini and the primary organoids was partially reverted by treatment with the Rho/MRTF/SRF pathway inhibitor CCG-203971. However, genetic activation of MRTF-A in the mature primary acini also reduced protrusion formation on stiff matrices, whilst it strongly promoted luminal filling matrix-independently.

**Conclusion:**

Our results suggest an intricate crosstalk between matrix stiffness and MRTF-A, whose activity is required for protrusion formation and sufficient for luminal filling of mammary acini.

**Video Abstract**

**Supplementary Information:**

The online version contains supplementary material available at 10.1186/s12964-022-00977-2.

## Highlights

- Formation of mammary acini depends on crosstalk between matrix stiffness and MRTF-A
- Increased matrix stiffness elevates MRTF-A activity and protrusion formation
- Protrusion formation of MCF10A-derived and primary murine acini is MRTF-dependent
- Genetic MRTF-A activation in primary organoids is sufficient for luminal filling

**Supplementary Information:**

The online version contains supplementary material available at 10.1186/s12964-022-00977-2.

## Background

The female mammary gland consists of epithelial cells with different functions and the surrounding stroma. The epithelial cells form a branched duct system and a lobuloalveolar system with several acini (alveoli) at the distal ends of the ducts [1]. Characteristic differentiation processes take place during sexual development and reproduction, and defective remodelling is thought to be causative for tumorigenesis. In particular, the loss of baso-apical polarisation in epithelial cells, which is crucial for the formation of the acinar lumen, is an important marker for malignancy and occurs during early tumorigenesis (reviewed in [2, 3]. In addition, hyperproliferation, irregular acinar shape and acinar outgrowth characterise pre-malignant transformation [4].

Independent of other factors, mechanistic stimuli of the extracellular matrix (ECM) are thought to promote pre-malignant morphological changes and breast cancer progression (as reviewed in [5]). Under physiological conditions mammary epithelial cells are subjected to varying mechanical stiffness ranging from normal soft mammary tissue characterised by a Young’s elastic modulus of 0.1–0.2 kPa to less compressive breast tumour tissue with approx. 4 kPa [4]. Areas of extreme mammographic density are associated with an increasing breast cancer risk especially in women aged 40–49 years [6–8]. A stiffer ECM induces a malignant phenotype in benign MCF10A-derived breast epithelial acini in vitro [4]. Analysing the regulation and induction of an undifferentiated malignant phenotype of mammary epithelial cells by altered mechanical properties of the ECM has become a major topic in breast cancer research, but the mechanistic details are still largely undefined [9–12].

One pathway implicated in mechanotransduction is the Rho/Actin/MRTF pathway [13–15]. The myocardin-related transcription factors (MRTF) A and B have a key role in cell differentiation, adhesion and tissue homeostasis, in conjunction with their basal transcription factor SRF (serum response factor) [16–21]. MRTF drives experimental metastasis and colonization by breast cancer cells [22]. Tightly controlled MRTF activation is required for the regular formation of acini in three-dimensional MCF10A acinar cultures on soft matrices, since overexpression of MRTF-A or MRTF-B leads to enlargement of acini, irregular shape of acini and lumen filling due to disturbed apico-basal polarity [23]. Furthermore, transcriptional activity or expression of MRTF-A increases in response to a stiff ECM in normal murine mammary gland epithelial cells [24], human colonic myofibroblasts [25], human adipose-derived stem cells [26] or osteosarcoma cells [27]. In the murine breast, MRTF-A knockout provokes development of larger and less organised lactating mammary glands, and defective myoepithelial cell differentiation [28, 29].

Given the increasing evidence for an oncogenic function of mechanosensitive MRTF in the control of acini genesis, we investigated how extracellular matrix, physical stiffness and MRTF-A activity interact in disrupting acinar formation and maintenance. We used MCF10A-derived acini as well as primary acinar organoids derived from mammary glands of nulliparous mice [3, 30–32]. Morphological changes indicative of premalignant transformation, such as loss of the hollow lumen in mature acini or outgrowth of acinar cells and formation of protrusions, were analysed. We confirmed that increasing matrix stiffness correlates with increased MRTF-A activity and enhanced protrusion formation. Inhibition of the Rho/MRTF/SRF pathway and ectopic high MRTF-A apparently reduces protrusions indicating that MRTF-A is required but not sufficient for protrusion formation. In addition, increased MRTF-A activity promotes luminal filling independent of matrix stiffness in mammary organoids.

## Methods

### Cell culture

MCF10A mammary epithelial cells (American Type Culture Collection, ATCC) were maintained in DMEM/F12 (Sigma-Aldrich) supplemented with 5% horse serum, 10 μg/ml insulin, 20 ng/ml epidermal growth factor (EGF), 100 ng/ml cholera toxin, 0.5 μg/ml hydrocortisone (all Sigma-Aldrich) at 37 ℃ and 5% CO_2_. For 3D culture MCF10A growth medium with 2% horse serum and 2% Matrigel (Corning) was used.

### Primary organoids

Nulliparous female NMRI mice (Charles River Laboratories) and female littermates of conditional HA-MRTF-A Δ3-5 transgenic mice (tg; manuscript in preparation) mated with heterozygous Rosa26Cre-ERT2 mice were maintained in accordance with the guidelines of the authorities in the local animal facility of the Martin-Luther-University of Halle-Wittenberg. Mice were sacrificed at 7–10 weeks of age, the mammary glands 2–4 were excised, visible lymph nodes were removed. Primary organoids were isolated as described previously [31, 33] with minor modifications. Briefly, the tissue was minced twice using the McIlwain Tissue Chopper (100 µm slices, 90° angle) following an incubation of 30–40 min in collagenase Type IV/Trypsin solution at 37 ℃. After multiple centrifugation steps, red blood cells were removed using red cell lysis buffer (Sigma). DNase I digestion was performed by using 2 U/ml DNase I (Sigma-Aldrich) for 10 min. Organoids were washed and the final pellet was resuspended with DMEM/F12 (PAN Biotech) without L-valine supplemented with D-valine, L-glutamine, 5% fetal calf serum (FCS, both Life Technologies), 0.01% insulin transferrin sodium selenite (ITS, Sigma), 0.05 mg/ml gentamycin (Thermo Scientific) and 2% Matrigel (Corning) and cultivated in 6-well plates for 30–40 min to remove fibroblast. Afterwards organoids were collected, centrifuged and seeded on 3D ECM gels. Treatments with the vehicle control (DMSO), 0.25 µM 4-OH-tamoxifen or 20 µM CCG203971 (Tocris Bioscience) were performed 16–36 h after seeding as indicated per experiment.

### 3D gel preparation and cell cultures

Three-dimensional collagen I matrix was prepared from 8 parts FibriCol (approx. 10 mg/ml; Advanced Biomatrix), 1 part 10 × PBS, 0.5 parts 0.1 M NaOH and 0.5 parts sterile water. The ready-to-use collagen I mixture was carefully mixed with Matrigel (Corning) in indicated ratios (75:25, 50:50, 25:75) to obtain different gel stiffness. Fifty-five µl of the mixed Matrigel-collagen I matrix, Matrigel or collagen I alone were gently spread per well of an eight-well chamber slide (Corning) following gelation at 37 °C for 1 h. For coating, 150 µl of a 1:10 diluted collagen IV solution (0.3 mg/mL, BioReagent) were added per well. After 90 min at 37 °C the solution was removed and the 3D gels rinsed with sterile Hanks ‘ Balanced Salt Solution (HBSS).

The matrix was then overlaid with 5000 MCF10A cells per well or primary organoids in 400 µl 3D culture medium. Synthetic polyacrylamide gels (12-well format, Matrigen SoftSlip) were covered with 2% Matrigel in PBS for 30 min at 37 ℃ before being overlaid with 100,000 MCF10A cells per well. For 3D morphogenesis assays MCF10A were cultured for max. 14 days with change of medium every second day. Primary organoids were cultured for a maximum of 6 days.

### Immunofluorescence and microscopy

The 3D MCF10A acini and primary organoids were fixed and stained as described [30]. The following primary and secondary antibodies were used: anti-laminin V (1:200, abcam #ab14509), anti-HA (1:200, Sigma #H6908), Alexa 546-donkey anti-rabbit (1:200, Invitrogen), Alexa-647-goat-anti-rabbit (1:200, Invitrogen). DNA was visualised using DAPI (Sigma) and F-actin was stained with phalloidin-atto 488 or phalloidin-atto 647 N (Sigma). Samples were imaged using a Zeiss Axio Observer 7 (Carl Zeiss Jena GmbH) equipped with apotome. Brightfield images of the acini were taken at a EVOS XL Core Microscope (Thermo Scientific). Pictures were analysed using the functions embedded in the Fiji software [34] to define diameter and roundness of acinar structures. Acinus diameter was measured in µm through the centre of each acinus from edge to edge at the widest point.

### Genotyping

Genotyping was performed from ear excisions or 5 day old primary organoids. After lysis in 400 µl lysis buffer (100 mM Tris–HCl, 5 mM EDTA, 0.2% SDS, 200 mM NaCl, pH 8–8.5) supplemented with 2 µl Proteinase K (New England Biolabs), DNA was precipitated with isopropanol. DNA concentration was determined using NanoDrop ND-1000 (NanoDrop) and 150 ng were used for subsequent PCR analysis using specific primers for the detection of the CreERT2-insertion (cre-fw 5’-AACATGCTTCATCGTCGG-3’ and cre-rv 5’-TTCGGATCATCAGCTACACC-3’) and for the analysis of the floxed STOP-cassette (floxed-fw 5’-GGCAACGTGCTGGTTATTGT-3’, floxed-rev 5’-GGACTCCACAGGCAGGATATT-3’). The PCR products were separated on a 1% agarose gel and imaged using the Gel stick system (Intas Science Imaging).

### mRNA expression analysis

RNA was extracted from acini using the Qiagen RNeasy Mini Kit (Qiagen) according to the manufacturers’ protocols including an on-column DNase digest. For cDNA synthesis the Verso cDNA Synthesis kit (Thermo Scientific) was used with 1 μg of total RNA and Oligo-dT primers. Transcript analysis was performed using the LightCycler 480 System (Roche, Mannheim Germany) and DyNamo Colorflash SYBR Green qPCR kit (Thermo Scientific) according to the manufacturer’s instructions using gene specific primer for GAPDH (5′-ACCCAGAAGACTGTGGATGG-3′; 5′-TTCTAGACGGCAGGTCAGGT-3′), ACTA2 (5 ‘-CGGTGCTGTCTCTCTATGCC-3 ‘; 5 ‘-AGCAGTAGTAACGAAGGAATAGCCA-3 ‘), ALAS (5′-CTGCAAAGATCTGACCCCTC-3 ‘; 5′-CCTCATCCACGAAGGTGATT-3 ‘), and vimentin (5′-CCCTCACCTGTGAAGTGGAT-3 ‘; 5′-TCCAGCAGCTTCCTGTAGGT-3 ‘). Relative expression was determined using the 2^−ΔΔCt^ cycle threshold (Ct) method normalized to ALAS and GAPDH expression.

### Luciferase promoter reporter assay

Per experiment 300,000 MCF10A cells were transfected with 800 ng p3D.A-Luc firefly luciferase reporter construct and 200 ng pRL-TK renilla luciferase control construct, using Polyethylenimin (PEI). The following day cells were seeded in a 12-well-plate on readily prepared 3D gels. After three days the cells were lysed and luciferase activity measured using the Dual-Luciferase Reporter-Assay System Kit (Promega). Firefly luciferase signals were normalised to Renilla luciferase activity and control transfected cells.

### Statistical analysis

Acini with protrusions, defined as cellular outgrowth of single or multiple cells exceeding the laminin-rich layer surrounding the acini, were individually scored with “100”, and “0” in their absence. Acini with partial or complete filling of the acini were scored with “100”, whereas those whose lumen remained hollow were scored with “0”. As indicated, multiple acini or organoids were analysed per experiment. Percentages were calculated as [%] = [sum of scores] / [number of scored acinar structures]. For MCF10A-derived acini percentages per seeded passage were combined for statistical analysis. Primary organoids were analysed independent of the mouse of origin. Graph Pad Prism 7 was used to generate all graphs and to perform statistical analysis. Data are presented as mean ± SEM, if not indicated different. Statistical analysis were performed using Student’s t-test, a one-way or a two-way ANOVA with Tukey's, Sidak's or Dunnett's multiple comparisons test, where *p* < 0.05 was considered statistically significant, and p values are denoted as follows: *p < 0.05, **p < 0.01 and ***p < 0.001.

## Results

### High matrix stiffness disturbs regular MCF10A acini formation and promotes protrusions

**Fig. 1:**
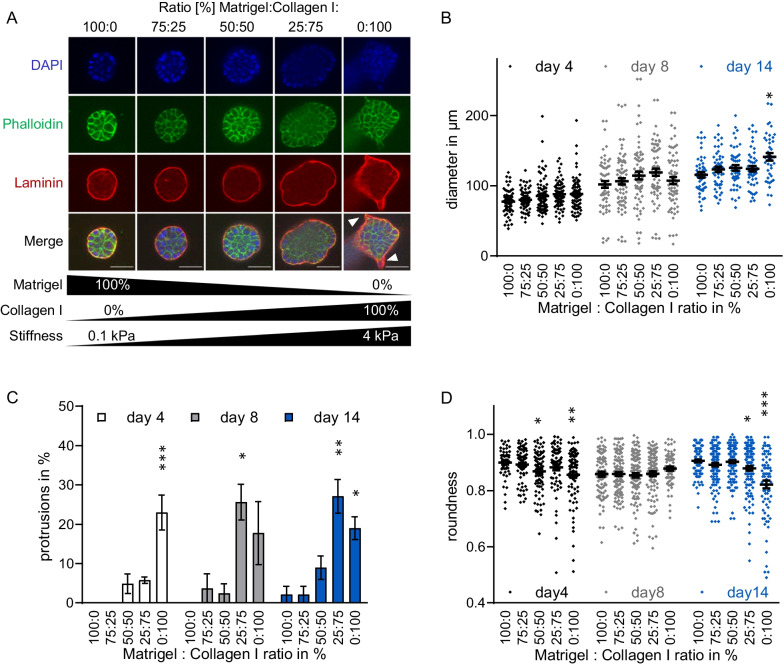
Correlation of increasing matrix stiffness with protrusion formation of MCF10A acini. 3D cultures of MCF10A cells on the indicated Matrigel/Collagen I mixtures (100:0, 75:25, 50:50, 25:75, 0:100) were analysed by microscopy. **A** Phalloidin (green), Laminin V (red) and nuclei (DAPI, blue) staining of acinar structures at day 4 after seeding on matrices with increasing Collagen I percentages. Arrowheads indicate protrusions. The corresponding increase of Young's elastic modulus of the matrix is depicted below. **B** Diameters of individual acini (diamonds) measured at day 4, day 8 and day 14. Black horizontal lines indicate mean diameter and SEM (whiskers) of three biologically independent replicates. **C** Quantification of protrusion formation at day 4, day 8 and day 14. At least 11 acini were assessed each day, the percentage of protrusion-positive acini calculated, and the entire experiment repeated 3 times. **D** Calculated roundness index of individual acini (diamonds). Statistical significance compared to acini formed on pure Matrigel was determined using a one-way ANOVA and the means for each time point. *p ≤ 0.05, **p ≤ 0.01, ***p ≤ 0.001. *Error bars*, SEM (*n* = 3). *Scale bars*, 50 μm

Since acinar morphology has been shown to be affected by both MRTF activity and tissue stiffness, we wanted to assess whether they cooperate during acini formation. First, we analysed changes in three-dimensional acinar morphogenesis induced by various matrix compositions of different stiffness. We thus cultured human mammary MCF10A epithelial cells on solidified Matrigel, Matrigel/collagen I mixtures (75%/25%, 50%/50%, 25%/75%) and pure collagen I. The increasing collagen I content leads to increasing stiffness of the solidified matrix with an elastic Young's modulus from about 0.1 kPa for soft Matrigel to about 4 kPa for stiffer collagen I [4]. To enable 3D acini formation, the overlay medium contained a constant amount of 2% matrigel in all conditions. The stiffness-dependent effects on the size and shape of the acini were analysed on day 4, 8 and 14 after cell seeding. Immunofluorescence analysis revealed an increasingly irregular shape and single- or multi-cellular outgrowth already in early 3D acinar structures on stiffer matrices (Fig. 1A). The average acini diameter, however, remained comparable, regardless of matrix composition (Fig. 1B). A moderate and rather expected increase of the acini diameter was observed correlating with the cultivation duration.

Quantitative analysis showed that stiffer collagen I-rich matrices (75–100%) promote significant protrusion formation independently of the cultivation time (Fig. 1C), consistent with previous observations [4]. For example, protrusion formation on collagen I increased by 23%, 17.8% and 17.9% compared to Matrigel on day 4, day 8 and day 14, respectively. Protrusion formation was accompanied by a more irregular acini shape as demonstrated by the significantly lower roundness of acini grown on Matrigel compared to those cultured on 75–100% collagen I (Fig. 1D).

Similar morphological changes were observed for human MCF7 breast cancer cells cultured on solidified Matrigel/collagen I mixtures for 4 days (Additional file 1: Fig. S1). The average diameter of the MCF7 spheroids remained constant independent of matrix properties. Also in this cell line, high collagen I content in the culture matrix promoted acinar outgrowth. Compared to soft Matrigel layer, 4.2–13.4% of the spheroids grown on matrix mixtures containing 50%, 75% or 100% collagen I developed protrusions.

### Stiffness but not underlying matrix composition governs acini formation

Increasing the percentage of collagen I in the base layer does not only change the stiffness of the matrix, but also its molecular composition and thereby, potentially, the ligands available for cellular receptors such as integrins. Thus we investigated whether the observed morphological changes were mediated by the increased collagen I content or by the increased stiffness of the underlying matrix. For this purpose, we firstly analysed the formation of MCF10A acini on Matrigel (MG) and collagen I (COL1) matrices, which were both thinly precoated with collagen IV (COL4), a constituent component of the basal lamina adjacent to mammary acinar structures.

**Fig. 2:**
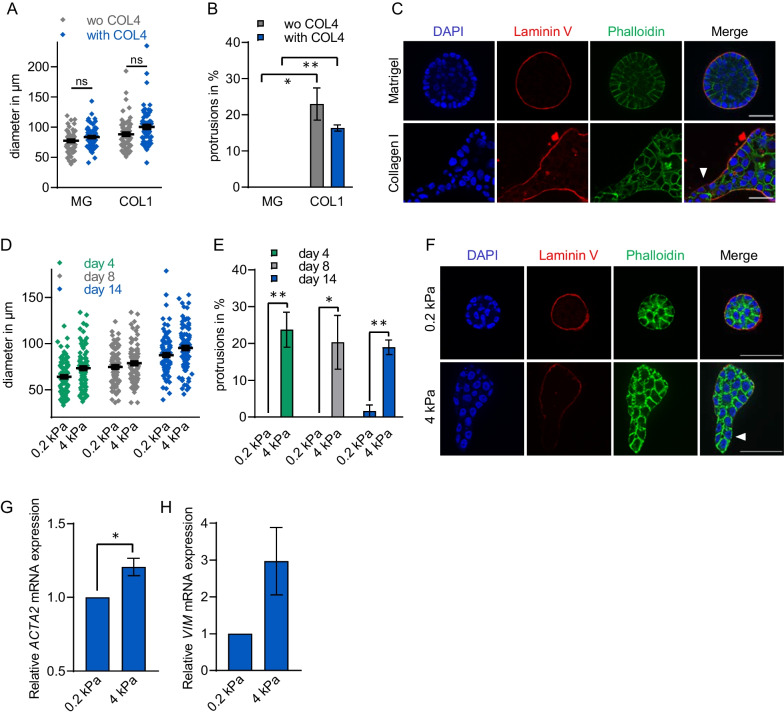
Acinar protrusions are independent of composition but depend on stiffness of matrix. **A**, **B**, **C** Matrigel (MG) or collagen I (COL1) gels were thinly coated with collagen IV (COL4) or left uncoated (wo COL4) as indicated. Subsequently MCF10A cells were seeded on top and acinar diameter **A** and protrusion formation **B** was analysed 4 days post seeding. **C** Representative micrographs of acini stained with laminin V (red), actin filaments with phalloidin (green) and cell nuclei with DAPI (blue) on day 4 of acini morphogenesis. Arrowheads indicate protrusions. **D**, **E**, **F** MCF10A cells were seeded on pre-cast acrylamide gels with defined stiffness of 0.2 kPa and 4 kPa coated with Matrigel, respectively. Acini diameter **D** and average percentage of protruding acini (**E**) were determined 4, 8 and 14 days post seeding as before. **F** Representative micrographs of acini on day 4 after seeding on the matrix indicated. Statistical significance was determined by ANOVA using the means of 3 independent experiments, each analysing ≥ 22 acini per condition. **G**, **H** Relative mRNA expression of ACTA2 **G** and VIM **H** normalised to ALAS and GAPDH. Statistical significance according to an unpaired Student’s *t*-test (*n* = 4). *p ≤ 0.05, **p ≤ 0.01. *Error bars*, SEM. *Scale bars*, 50 μm

Collagen IV coating did not significantly alter the mean diameter of MCF10A acini grown on either Matrigel or collagen I matrices for 4 days, as compared to the uncoated matrix (Fig. 2A). As before, protruding acini significantly increased on the stiffer COL1 base layer, but additional precoating with COL4 did not result in a significant reduction of protrusion formation (Fig. 2B). Rather, fluorescence microscopy revealed profound differences of the acini grown on Matrigel or collagen I, even though both were coated with collagen IV (Fig. 2C). This suggests that it is not the presentation of collagen I on the matrix which promotes protrusion formation.

In a second experimental approach, we seeded MCF10A cells onto base layers of commercially available pre-cast acrylamide gels with defined stiffness of 0.2 kPa and 4 kPa. Both gels were coated with Matrigel to allow cell adhesion. Diameter and shape of the acini were examined 4, 8 and 14 days after cell seeding. The results confirmed that an increased stiffness of 4 kPa of the underlying matrix did not significantly change the diameter of the acini structures formed (Fig. 2D). However, formation of protrusions was triggered almost exclusively on the pre-cast acrylamide gels with high stiffness (Fig. 2E and F). These results suggest that the role of particular matrix components or diffusable ligands can be neglected for protrusion formation on collagen I matrices under our experimental conditions, but that physical stiffness causes the increased protrusion formation of MCF10A acini.

To corroborate the stiffness-induced malformation of acini with mammary epithelial characteristics, we analysed the expression of the markers smooth muscle alpha actin (ACTA2) and vimentin (VIM). mRNA was collected from acini grown for 4 days on the defined "soft" acrylamide gels with 0.2 kPa, or from those with 4 kPa which permitted protrusion formation in around 15% of acini. Both markers were slightly upregulated in acini grown on the stiffer acrylamide gels with 4 kPa (Fig. 2G and H). Whilst ACTA2, a known MRTF target gene, was only induced by around 20 percent, the mesenchymal marker VIM was non-significantly elevated threefold, suggesting that the cells are changing their luminal epithelial fate when growing on stiffer matrices.

### MRTF/SRF is required for stiffness-dependent protrusion formation

**Fig. 3:**
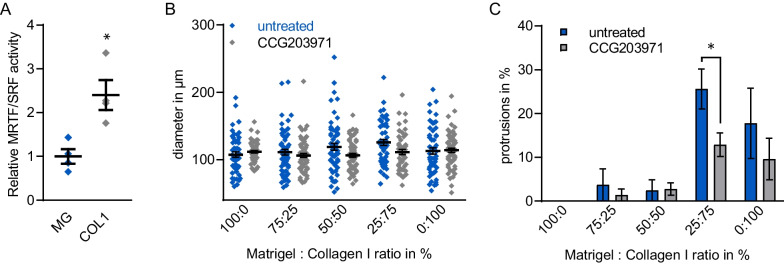
Implication of the MRTF-SRF pathway in stiffness-dependent acini morphogenesis. **A** Relative MRTF/SRF luciferase reporter activity in transiently transfected MCF10A seeded on Matrigel (MG) or collagen I (COLI) and analysed at day 3 of acini morphogenesis (*n* = 4). **B**, **C** Effects of the Rho/MRTF/SRF pathway inhibitor CCG203971 (20 μM, day 4–8) on acini diameter and average percentage of protrusion-positive acini at day 8. Eleven or more acini were analysed per condition in 3 biologically independent replicates. **B** Individually measured acini diameter (diamond) and mean ± SEM (horizontal black line ± whiskers). **C** Percentage of protrusion formation. Significance was tested using an unpaired Student’s t-test. *p ≤ 0.05. *Error bars*, SEM

After having validated our model of matrix stiffness, we next analysed MRTF/SRF activity in MCF10A acini grown on soft and stiff ECM gels. Using a promoter-luciferase reporter construct [35] dependent on transcriptional activation by the MRTF/SRF complex, we observed a moderate but significant increase in MRTF/SRF activity in 3D acini grown on stiff collagen I matrices for three days, compared to those grown on soft Matrigel (Fig. 3A). Accordingly, we hypothesised that activation of MRTF-A dependent transcription during acinar formation on stiff matrices might contribute to irregular spheroid formation and development of protrusions. We thus treated 3D acini cultures from day 4–8 during morphogenesis with the Rho/MRTF/SRF pathway inhibitor CCG203971 and analysed the morphological changes on matrices with increasing stiffness. Compared to the untreated control, CCG203971 did not significantly alter the average diameter of the acini regardless of the stiffness of the underlying matrix (Fig. 3B). However, the average protrusion formation of acini treated with CCG203971 was reduced by approximately 50% on stiffer collagen I matrices, exhibiting statistical significance on a Matrigel/collagen I mixture of 25:75 (Fig. 3C). Reduced protrusion formation correlated with reduced MRTF/SRF reporter activity following CCG203971 treatment (Additional file 1: Figure S2). Together, these results suggest a correlation of high MRTF/SRF activity and stiffness-dependent protrusion formation in 3D acini of MCF10A cells.

### Matrix stiffness and MRTF activity cooperatively affect primary organoids

**Fig. 4:**
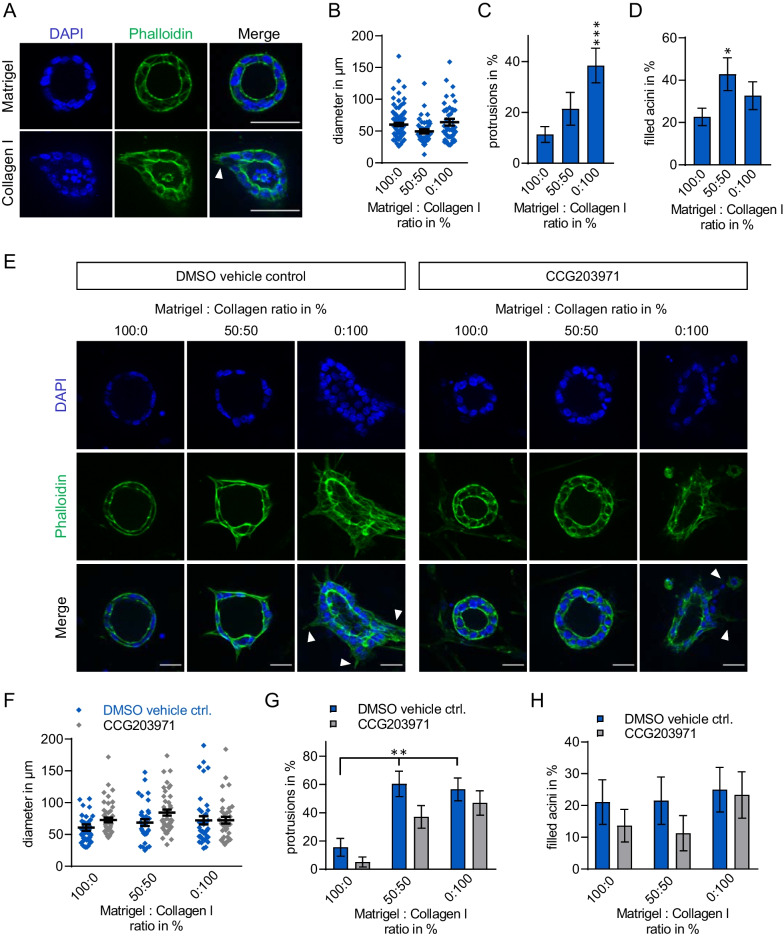
Matrix stiffness and MRTF/SRF activity cooperate on protrusion formation in mature murine acini. Acinar organoids were extracted from the mammary gland of 8–12 week old female NMRI mice. Organoids were seeded on matrices with increasing stiffness, and morphologies were analysed 5 days after seeding by microscopy. **A** Representative images of primary murine acini stained for nuclei (DAPI, blue) and actin (phalloidin, green) on matrigel or collagen I matrices 5 days after seeding. Arrowheads indicate protrusions. *Scale bars*, 50 μm. Individual acini diameter **B**, percentage of protrusion-positive acini **C** and percentage of filled acini **D** were quantified (≥ 42 individual acini per condition, one-way ANOVA). (**E**) Representative images of acini after 4 days of treatment with DMSO or 20 μM CCG203971. *Scale bars*, 25 μm. Individual acini diameter **F**, percentage of protrusion-positive acini **G** and percentage of filled acini **H** (≥ 24 individual acini per condition, two-way ANOVA). *p ≤ 0.05, **p ≤ 0.01, ***p ≤ 0.001. *Error bars* or *whiskers*, SEM

In vivo, the mature lobulo-alveolar system is more complex, and acinar structures are already pre-existing. To partially account for the increased complexity and to further analyse stiffness-mediated effects on the mature acinus, we extended our experimental approach to primary acini from 8 to 12 week old female NMRI mice. Epithelial mammary acini were extracted and cultivated on 3D matrices for 5 days, followed by microscopical analysis of the acinar structures. When grown on soft Matrigel, organoids appeared round and hollow, whereas organoids on stiff collagen I gels lacked regularly shaped acinar morphology (Fig. 4A). As with MCF10A-derived acini, no stiffness-induced alteration of the diameter of the mature murine acini was observable. In contrast, increasing matrix stiffness correlated with significantly increased formation of protrusions (Fig. 4B, C). We also analysed luminal filling of the preformed acinar structures, but quantification of more than 40 organoids revealed only a minor increase of filled acini on stiffer matrices under these experimental conditions (Fig. 4D).

Subsequently we analysed whether the MRTF/SRF pathway and matrix stiffness functionally cooperate in protrusion formation, using the inhibitor CCG203971. As before, fluorescence microscopy revealed increasing protrusions and irregular morphology after mature acini were grown for 5 days on matrices with gradually increased stiffness. Vehicle control acini showed protrusions already on 50:50 mixtures of matrigel and collagen, which was partially reverted by CCG203971 treatment at this stiffness (Fig. 4E). No profound change in the diameter of CCG203971-treated organoids was measured compared to the vehicle control acini (Fig. 4F). Quantification confirmed a strong trend towards a CCG203971-mediated reduction of protrusion formation of preformed acini on matrices with intermediate stiffness. However, this inhibition seems to be overcome by cultivating the organoids on 100% collagen. Regarding filling of the acinar lumen, only a slight and non-significant effect of CCG203971 treatment was observed upon quantification of hollow structures under these conditions (Fig. 4H).

### Overactivation of MRTF-A causes luminal filling regardless of matrix stiffness

Finally, we utilised a genetically encoded mouse model for an inducible gain-of-function of MRTF-A to analyse how MRTF-A affects primary murine acini cultured on soft and stiff matrices. The Tamoxifen-inducible CreERT2/loxP system was used to generate a conditional knockin of constitutively active MRTF-A in C57BL/6NCrl mice (A.K.S. and G.P., manuscript in preparation). The encoded MRTF-A lacks the RPEL1/2 motifs required for G-actin binding and repression. As described above, organoids were extracted from mammary glands of 8–12 week old transgenic mice which allow (tg) or not allow (wt, negative for CreERT2) for the expression of activated MRTF-A upon treatment with 4-hydroxy-tamoxifen (4OHT).

**Fig. 5:**
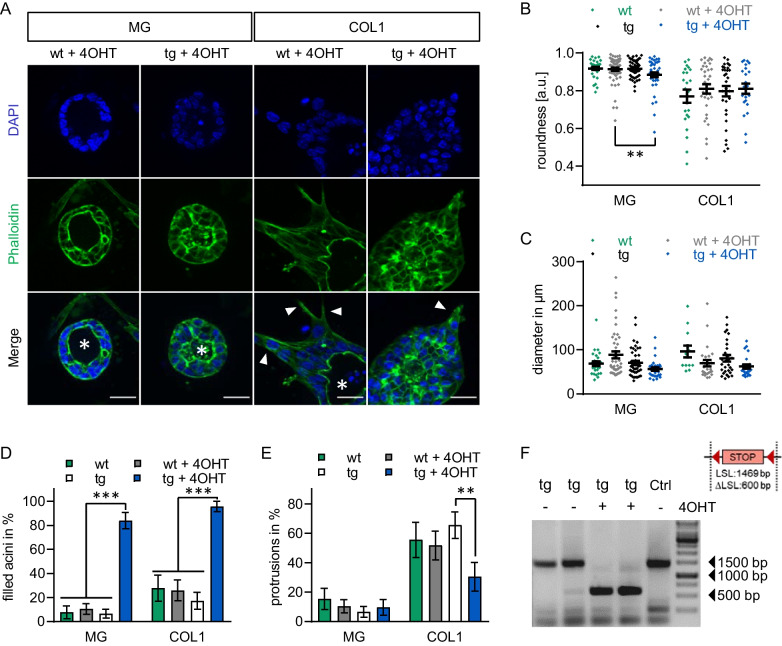
High MRTF-A activity causes luminal filling in primary mammary acini. Acinar organoids were extracted from the mammary gland of 8–12 week old female wildtype (wt) and transgenic (tg) mice carrying a LSL-MRTF-A cassette and CreERT2. Organoids were seeded on matrices with increasing stiffness and treated for 4 days with 0.25 μM Hydroxy-Tamoxifen (4OHT) or vehicle control. **A** Representative images of primary murine acini stained for nuclei (DAPI, blue) and actin (phalloidin, green) on matrigel/collagen I matrices 5 days after seeding. Arrowheads indicate protrusions, and asterisks the acinar lumen. **B**, **C** Distribution of individual organoid roundness and diameters. **D**, **E** Percentage of acini with filled lumen and protrusions. More than 17 acini were analysed per condition. Significance was determined by two-way ANOVA. **F** Genotyping of 4OHT-treated ( +) and vehicle treated ( − ) tg acini using primer flanking the loxP STOP loxP cassette (LSL, 1500 bp) in comparison to an untreated positive control sample (Ctrl). *p ≤ 0.05, **p ≤ 0.01, ***p ≤ 0.001. *Error bars* or *whiskers*, SEM. *Scale bars*, 25 μm

Organoids were treated with a vehicle control or 4OHT 24 h post seeding to initiate expression of activated MRTF-A. After 4 days of incubation, acini were fixed, stained and morphological changes were analysed. The stiff collagen I matrix lead to irregularly shaped organoids and the formation of protrusions, compared to round control acini cultured on the soft Matrigel (Fig. 5A, B). Strikingly, overactivation of MRTF-A by 4OHT treatment of tg organoids strongly promoted luminal filling, irrespective of the matrix stiffness. Quantification revealed that acini roundness was reduced on stiff matrices, whilst the average diameter of the organoids was largely unaffected, as before (Fig. 5B, C). The stiffer matrix slightly elevated the rate of luminal filling also in control organoids, consistent with previous reports [4], but this was strongly exacerbated in 4OHT-treated tg organoids showing a profound loss of hollow acinar structures (Fig. 5D). The number of protrusion-forming organoids was again around three times higher on the stiffer matrix (Fig. 5E). Interestingly, however, MRTF-A activation did not induce protrusion formation on matrigel, and even inhibited it on the stiff matrix, compared to untreated tg and wt acini. These results suggest that high MRTF-A activity is sufficient to cause luminal filling of preformed mammary acini, but that it is insufficient to promote protrusion formation.

Although the increase of MRTF-A activity could not be quantified due to the minute amount of cell material available, genotyping of tg acini after 4 days in the presence or absence of 4OHT confirmed the removal of the STOP cassette via the ERT2-controlled Cre recombinase (Fig. 5F). Primary murine tg and wt acini were also cultured on matrices of different stiffness for three days to allow recovery and subsequently treated with 4OHT for two days before microscopic examination. Here, staining of HA-labelled MRTF-A provided evidence of expression of the activated MRTF-A transgene in acini after 4OHT treatment (Additional file 1: Fig. S3A). Moreover, even the shorter expression of activated MRTF-A was sufficient to significantly promote luminal filling without causing protrusion formation on soft matrix (Additional file 1: Fig. S3B, C). On the stiff matrix, however, MRTF-A overactivation rather inhibited protrusion formation, in line with a previously proposed uncoupling from mechanotransduction [23].

## Discussion

The mammary gland is embedded in situ in an extracellular matrix (ECM) composed predominantly of laminin (laminin-111, laminin 332, laminin-511 and laminin-521) and collagen IV, while small amounts of fibronectin, tenascin and collagen I are present [36]. The reconstituted basement membrane preparation Matrigel resembles this composition with approximately 60% laminin and 30% collagen IV and further mimics the stiffness of soft normal breast tissue with a Young’s Elastic modulus of 0.1–0.2 kPa [4]. Accordingly, the in vitro cultivation of acini on solidified Matrigel in Matrigel-supplemented Medium is one of the most standardised 3D models for analysing effectors causing pre-malignant mammary transformation [2].

In the mammary gland structure, the individual ECM proteins are unevenly distributed. For example, a laminin- and collagen IV-rich basal membrane (BM) mainly surrounds lobuloalveolar structures, while collagen I is most abundant around the large milk ducts. At their sites of action, the ECM components interact with epithelial cells to maintain either ductal or acinar structures, or to mediate morphological changes [4, 9]. Especially increased stromal collagen I deposition promotes mammary tumorigenesis in transgenic murine model and is associated with increased mammographic density, which is an established risk factor in breast cancer [6, 11, 37]. To mimic the physiological conditions and analyse stiffness-dependent effects, we used solidified Matrigel/collagen mixtures in the established MCF10A-based acini formation model, whereby the addition of Matrigel to growth medium promoted the formation of a laminin-rich BM as confirmed via immunofluorescence (Fig. 1). The usage of collagen I allowed us to alter the rigidity of the underlying matrix in the physiological range from normal until fibrotic and/or tumorous breast tissue with a Young’s Elastic modulus of 3–4 kPa [4]. An increased collagen I density promotes in vitro a more invasive and proliferative phenotype in mammary epithelial cells [4, 38]. In line with these results, we observed a less rounded acini structure and increased protrusion formation in normal MCF10A- and cancerous MCF7-derived epithelial organoids grown on stiffer matrices with increasing collagen I content. A thin coating with Collagen IV on pure collagen I gels or a thin Matrigel-coating on synthetic acrylamide gels of defined stiffness did not substantially change these observations (Fig. 2). Although a minor effect of pore size or biochemical composition cannot be ruled out, the stiffer matrices caused an increase of MRTF-A activity (Fig. 3), as reported by others in multiple cell types [24–27].

### The role of the MRTF/SRF pathway in protrusion formation

While the immortalised cell lines were exposed to various mechanical stimuli during the course of acini formation, primary organoids extracted from fully developed murine breast tissue allowed us to study matrix-induced effects in vitro on mature organoids. This is physiologically relevant as the development of breast carcinoma likely begins in the adult gland, whose development is completed at the end of puberty. In addition, the primary organoids are partially surrounded by their natural niche of myoepithelial cells and fibroblasts. On the other hand, extraction from individual mice results in increased variability, which is also reflected in the data presented. As shown for MCF10A- and MCF7 derived acini, a significant increase in protrusion formation on firmer matrices could be demonstrated in mature organoids of wildtype NMRI mice (Fig. 4). Moreover, inhibition of the MRTF-A signalling pathway with the chemical Rho/SRF/MRTF pathway inhibitor CCG203791 caused a reduction in protrusion formation, similar to that observed in MCF10A-derived acini. In this context, the moderate reduction of MRTF-A activity by CCG203791 treatment of preformed acini had only a weak effect on lumen formation and none on acinar diameter, in contrast to the grossly disorganised acini with reduced size and luminal filling we reported previously for MRTF-A knockdown MCF10A cells [23]. However, the treatment with CCG203791 and further second generation Rho/MRTF/SRF pathway inhibitors previously reduced an invasive, migratory and tumorigenic phenotype also in pancreatic cancer [39], osteosarcoma [27] or human colonic myofibroblasts [25], which is in line with the reduced protrusion formation of primary murine and MCF10A-derived acini on stiffer matrices presented here.

### Stiffness-decoupled effects of increased MRTF-A activity

Our results imply a critical role of the SRF/MRTF-A pathway in protrusion formation. However, the constant expression of transcriptionally active MRTF-A did not promote protrusion formation in mature acinar organoids. Surprisingly, stiffness-induced protrusion formation was even reduced in the presence of a constitutively active MRTF-A construct lacking the inhibitory RPEL 1 and 2 motifs (Fig. 5 & Additional file 1: Fig. S3). Here, the activated HA-tagged MRTF-A was expressed in mature acini in vitro upon Tamoxifen-induced deletion of a STOP cassette in a ROSA26 transgene (A.K.S. and G.P., manuscript in preparation). By inducing aberrant transcription of target genes, overactivated MRTF-A likely uncouples mechanotransduction from the matrix, in line with our previous findings, and effectively reduced protrusion formation [23].

On the other hand, overexpression of MRTF-A strongly promotes luminal filling in mature primary organoids, as reported for MCF10A-derived acini formation (Fig. 5 & Additional file 1: Fig. S3) [23]. This effect was independent of the surrounding ECM. In wildtype organoids, no consistent ECM-induced effect on the maintenance of the mature organoid lumen was observed in our experimental conditions, which may post the question if stiffness initially induces luminal filling in mature acinar structures, as it does in many cell culture models, or rather initiates invasive protrusion formation. In general, filling of the lumen is considered as a pre-malignant lesion which is sequentially followed by epithelial to mesenchymal transition (EMT) facilitating invasion of carcinoma cells [40].

**Fig. 6:**
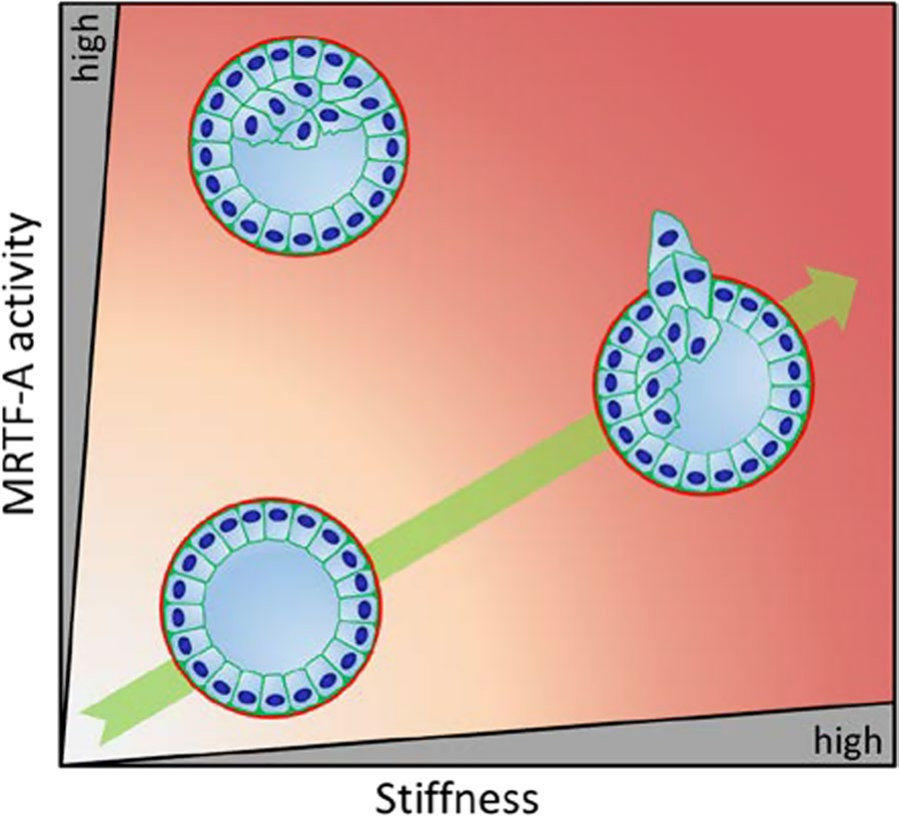
Model of the functional interplay between matrix stiffness and MRTF-A activity. Increasing matrix stiffness correlates with increased MRTF-A activity, enhanced protrusion formation and luminal filling. Alteration of the Rho/MRTF/SRF pathway reduces protrusions, indicating that MRTF-A is required but not sufficient for protrusion formation. In addition, increased MRTF-A activity promotes luminal filling independent of matrix stiffness in mammary organoids

However, our observational study suggests individual roles of MRTF-A in both processes (Fig. 6). While increased MRTF-A activity is required but maybe not sufficient for the formation of stiffness-induced protrusions, increased MRTF-A activity promotes luminal filling independently of the ECM. As shown previously in MCF10A, hyperproliferation upon MRTF-A activation may contribute to luminal filling of transgenic organoids, and also to protrusion formation [23]. Alternatively, but not mutually exclusive, cell fate changes characterised by expression of cytoskeletal components and EMT markers such as ACTA2 and vimentin may permit filling of the lumen and initiate protrusion formation. We speculate that these changes cooperate in perturbing distinct steps during formation and maintenance of hollow acinar structures. The specific molecular mechanisms governing these effects need to be explored.

## Supplementary Information

**Additional file 1: Fig. S1:** Analysis of matrix dependent effects on MCF7 spheroid formation. **Fig. S2:** CCG203971 reduces the relative MRTF/SRF activity but does not significantly alter cell viability of MCF10A cells grown in standard 2D cultures. **Fig. S3:** Two days exposure to high MRTF-A activity causes luminal filling and reduces protrusion formation in primary mammary acini cultures.

## Data Availability

The datasets used and/or analysed during the current study are available from the corresponding author upon reasonable request.
