# Supplementary material for: Interplay of the transcription factor MRTF-A and matrix stiffness controls mammary acinar structure and protrusion formation
Source: Cell Commun Signal. 2022 Oct 13;20:158. doi: 10.1186/s12964-022-00977-2 (PMC9563482; doi:10.1186/s12964-022-00977-2)
Supplement: Supplementary file 2 — Additional file 1: Fig. S1: Analysis of matrix dependent effects on MCF7 spheroid formation. Fig. S2: CCG203971 reduces the relative MRTF/SRF activity but does not significantly alter cell viability of MCF10A cells grown in standard 2D cultures. Fig. S3: Two days exposure to high MRTF-A activity causes luminal filling and reduces protrusion formation in primary mammary acini cultures. [file 12964_2022_977_MOESM2_ESM.pdf]

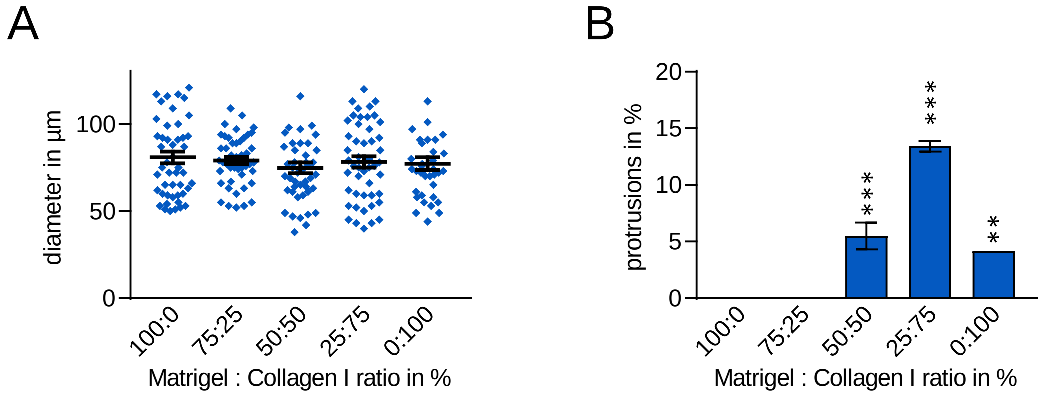

Supplementary Figure S1: Analysis of matrix dependent effects on MCF7 spheroid formation. The morphology of MCF7 spheroids cultured on solidified Matrigel/Collagen I mixtures (100:0, 75:25, 50:50, 25:75, 0:100) was analysed on day 4 post seeding. **(A)** Changing matrix stiffness did not alter spheroid size. Shown are diameters of individual acini (blue diamonds). **(B)** Increasing Collagen I percentages (50-100%) resulting in more rigid matrices promote protrusion formation. Displayed are the mean ± SEM (black line with whiskers) of 3 independent experiments. Significant deviation from spheroids formed on pure Matrigel was assessed using a one-way ANOVA test. **$p<0.01$, ***$p<0.001$

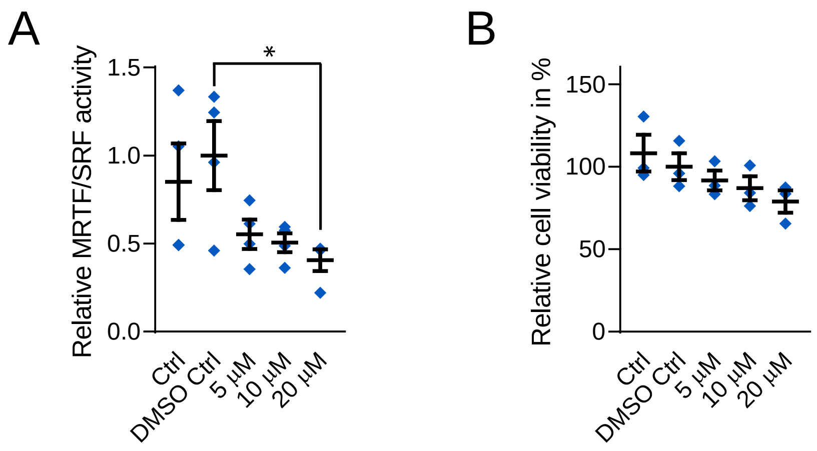

Supplementary Figure S2: CCG203971 reduces the relative MRTF/SRF activity but does not significantly alter cell viability of MCF10A cells grown in standard 2D cultures. (A) Relative MRTF/SRF activity of adherently grown MCF10A cells treated with increasing CCG203971 concentrations for 48 h. **(B)** Relative cell viability of MCF10A cells cultivated in 2D in the presence of increasing CCG203971 inhibitor concentrations after 48h. Shown is the mean ± SEM (black horizontal line with whiskers) of 3-4 independent experiments (blue diamonds). Significant differences in comparison to the DMSO treated control were determined using a one-way ANOVA with *p≤0.05.

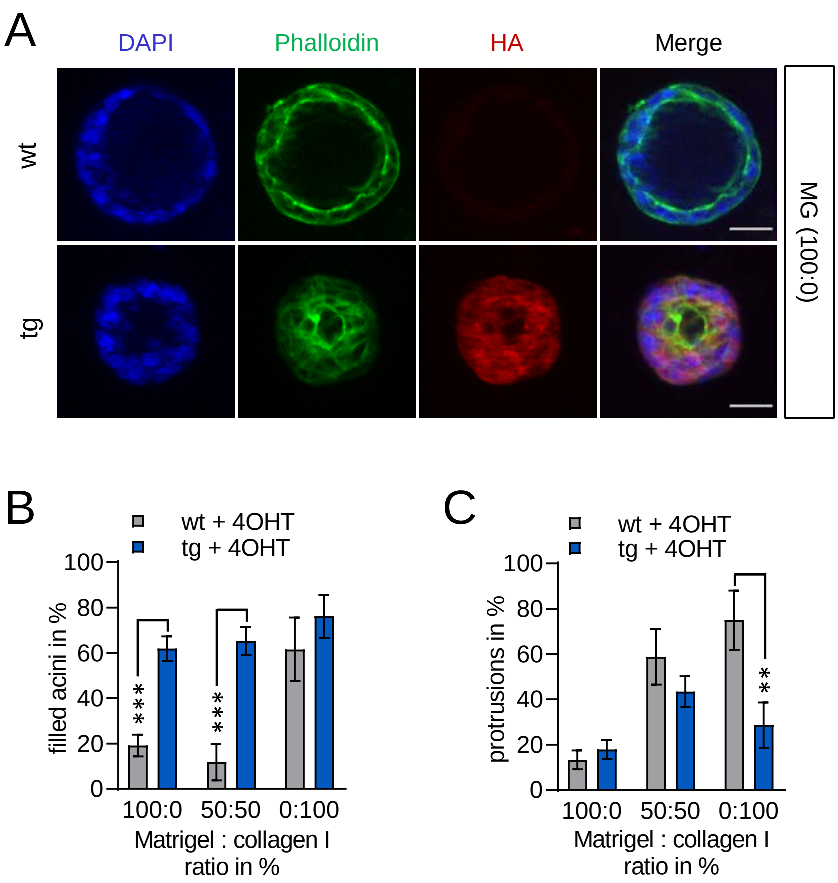

Supplementary Figure S3: Two days exposure to high MRTF-A activity causes luminal filling and reduces protrusion formation in primary mammary acini cultures. Acinar organoids were extracted from the mammary gland of 8-12 week old female tg and wt C57BL/6NCrl mice. Organoids were seeded on matrices with increasing stiffness and treated for 2 days with 0.25 µM 4OHT or a vehicle control 3 days post seeding. **(A)** Representative images of primary murine acini stained for nuclei (DAPI, blue), the HA-tagged MRTF-A Δ3-5 (HA, red) and actin (phalloidin, green) on matrigel. Scale bars represent 25 µm. The mean ± SEM of the percentage of filled **(B)** and protruding (C) acini are shown, with n≥12 individual acini analysed per condition. Significance was tested using a two-way ANOVA with **p<0.01 and ***p<0.001.
